# On the impact of incomplete taxon sampling on the relative timing of gene transfer events

**DOI:** 10.1371/journal.pbio.3002460

**Published:** 2024-03-18

**Authors:** Moisès Bernabeu, Saioa Manzano-Morales, Toni Gabaldón

**Affiliations:** 1 Barcelona Supercomputing Center (BSC), Barcelona, Spain; 2 Institute for Research in Biomedicine (IRB Barcelona), The Barcelona Institute of Science and Technology, Barcelona, Spain; 3 Catalan Institution for Research and Advanced Studies (ICREA), Barcelona, Spain; 4 Centro de Investigación Biomédica En Red de Enfermedades Infecciosas (CIBERINFEC), Barcelona, Spain

## Abstract

A recent study questioned the use of branch length methods to assess the relative timing of horizontal gene transfers because of the effects of so-called “ghost” lineages. This Formal Comment discusses key considerations regarding the potential effect of missing lineages when assessing relative timing of evolutionary events.

Processes of non-vertical evolution, such as horizontal gene transfer, drive genome evolution across life [1–3], resulting in gene trees incongruent with the species tree. Hence, gene phylogenies are used to infer non-vertical events, the lineages involved, and—more recently—their relative ordering [4]. A study by Tricou and colleagues [5] questioned the usage of branch length methods to assess the relative timing of transfers on the basis of so-called “ghost” lineages, as well as the validity of the conclusions of some studies, including from our group [6,7].

The existence of ghost (either extinct or unsampled) lineages relates to the well-known problem of data incompleteness. Evolutionary inference must be based on existing data, which is necessarily incomplete given, among other factors, incomplete sampling, pervasive extinction, and absence or scarcity of fossils. In this context, it is important to raise awareness of potential misleading conclusions that may arise from incompleteness, as Tricou and colleagues rightly do [5]. However, their discussion underemphasises the fact that incomplete sampling was addressed in several of the criticised papers, either by asking for caution [8] or by supplementary analyses [6].

Moreover, the simulations performed by Tricou and colleagues have some caveats that warrant further discussion [5]. Firstly, they use theoretical scenarios and parameters without considering current knowledge on the Tree of Life (ToL). Latest ToL reconstructions [9,10] (Fig 1A) show that the branch separating eukaryotes from their closest archaeal relatives (where the transfers in [6] occurred) is relatively short; in addition, the inferred bacterial donors in [6] branch deep in the bacterial phylogeny [9–11]. This scenario is very dissimilar from that in ***Fig 5*** by Tricou and colleagues [5]. Moreover, simulated topologies underlying ***Fig 6*** are unrelated to the conceptual scenario of ***Fig 5***, to the current ToL, or to the scenarios studied in [6,7]. Yet, they claim that ***Fig 6*** invalidates findings in [6,7]. We understand that models are inherently reductionist, but we believe they should be grounded on the proper context.

**Fig 1:**
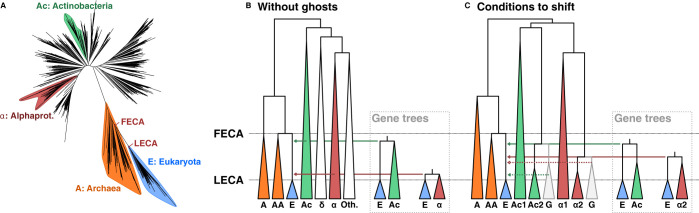
Collapsed species ToL from Hug and colleagues [9] and their expected gene trees with and without assuming ghost lineages. (A) Hug and colleagues [9] complete ToL with the groups studied in [6] highlighted. (B) At the left: schematic ToL tree with selected clades and with branch lengths taken from [9]. No ghost lineages are inferred. One recent transfer from alpha-proteobacteria and one ancient transfer from actinobacteria (Ac) are depicted by arrows (as observed in Pittis and Gabaldón [6]). Gene trees are depicted to the right. (C) Conditions needed to observe a shift. Two putative ghost donor lineages are assumed to result in the wrong acquisition timing classification for the donors (early and late). Dashed lines show the acquisitions from the ghost sisters of Actinobacteria and Alphaproteobacteria to eukaryotes, and solid lines show the distance we would obtain from the gene trees. In B and C, midpoint rooting was used, and the lengths of all internal branches were preserved as in A. Terminal triangular shapes indicating clades have been arbitrarily extended for visualisation purposes. AA: Asgard Archaea; A: Other Archaea; E: Eukaryotes; Ac: Actinobacteria; α: alphaproteobacteria; δ: deltaproteobacteria.

Let us take one of the claims made in [6,7]: that genes of alpha-proteobacterial origin are more recently transferred than those of actinobacterial descent (Fig 1B). To invalidate this claim, the ghost lineage would have had to branch very close to actinobacteria (to be assigned to that taxon), and would have transferred the gene more recently than the alpha-proteobacterial transfer (Fig 1C). It is feasible to assume that the real donors for both inferred transfers are long extinct (ghosts), but the set of constraints needed for these ghosts to be shift-inducing is rather restrictive, as several events have to occur: (a) the ghost has diverged inside the FECA-to-LECA period; (b) the ghost that transferred later has to have diverged before (earlier ghost) the ghost that transferred earlier (late ghost); and (c) the earlier ghost must have transferred after the later one. In essence, the ordering of the origin of the ghosts and of their transfer must have occurred in reverse.

The branch length of the inferred acquisition (i.e., the relative time point for the transfer) must be analysed in the context of the ghost’s divergence to its extant relatives and the history of the clade. For instance, although sensitive to extinction and speciation rates, earlier transfers are more likely to result from extinct lineages relative to later transfers, simply because more time has passed, resulting in longer branches. Conversely, later transfers are more likely to result from non-extinct lineages or from ghosts with closer extant relatives, as the ghost existed more recently. Thus, even assuming constant transfer and extinction rates, a ghost that transferred early and belongs to a deep lineage would result in longer gene tree branches for the early transfer, as the detected donor would be a farther ancestor, reducing, rather than increasing, the likelihood of a shift. These properties also make the results sensitive to the simulated tree topology (see below). Thus, in relation to the criticism of [6,7], to change the conclusion of the relative order of actinobacterial and alpha-proteobacterial transfers to the proto-eukaryote, the alpha-proteobacterial ghost would have had to have diverged after the supposed late actinobacterial acquisition (Fig 1C) in order for us not to be able to retrieve a close enough sampled relative of the ghost donor that provides the proper conclusion. This is a plausible but highly constrained scenario. In fact, this is one of the caveats that Susko and colleagues [12] discussed, and one that was actually addressed in the original analysis (see section 4 in the supplementary of [6]).

Finally, the speciation and extinction rates are treated as uniform through the simulations. We understand that modelling requires simplifications, but we feel that the importance of parameter choice has been understated by Tricou and colleagues [5]. The authors set an extinction rate of 0.9 for the *Anopheles* introgression [13] and D_3_ methodology [8], whereas 0.5 is used for the branch length ratio [6], which is non-trivial and is not justified in their article. Depending on the birth and death rates, the branch lengths differ, with higher death rates resulting in shorter terminal branches and lower ones in longer terminal branches [14] (Fig 2A). When using an extinction rate of 0.9, terminal branches are shorter (Fig 2B), as lineages tend to exist for less time. Moreover, as a consequence, the number of lineages against the root-to-tip distance (or time) curve slope increases at its end. Thus, removing some tips in these trees does not imply large changes in the topology of the deeper branches, making a shift to be less likely. However, if a lower extinction rate is used, then terminal branches are larger (Fig 2C) and the removal of tips disturbs in a higher degree the internal topology of the tree, causing shifts to be more likely.

**Fig 2:**
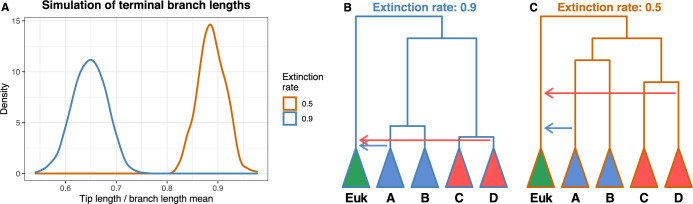
Impact of the extinction rate in timing conclusions. (A) Tip branch length distributions for each extinction rate are obtained from 1,000 simulated trees with 1,000 leaves each. Tip lengths are normalised according to the branch length mean distribution to have a relative measure of the tip length in relation to the entire tree. (B) Sample species tree using an extinction rate of 0.9. (C) Sample species tree using an extinction rate of 0.5. Data underlying this figure can be found in https://zenodo.org/doi/10.5281/zenodo.10234210.

We re-ran the analysis with the scripts provided on the supplementary material by Tricou and colleagues [5] over a range of extinction rate values and repeated ***Fig 6*** (see additional analysis at https://zenodo.org/doi/10.5281/zenodo.10234210). At an extinction rate of 0.9, and under the most stringent conditions for subsampling (1%), the percentage of incorrect predictions is 32.07% on average. In fact, the ratio of correct predictions increases when the trees are simulated using higher extinction rates, as we predict above. Moreover, in our run of the simulationhttps://zenodo.org/doi/10.5281/zenodo.10234210, an extinction rate of 0.5 yields a mean percentage of incorrect predictions of 40.55%. This is incongruent with ***Fig 6A***, which we could only replicate under a rate of 0.0 (incidentally, the value appearing in their code, see https://doi.org/10.5281/zenodo.6901799). These results were consistent across 24 runs per extinction rate.

In conclusion, we appreciate the efforts by Tricou and colleagues for addressing the possible confounding effects of unsampled lineages on evolutionary analyses, but we must strongly disagree with the extent to which they claim this affects previously obtained results. First and foremost, ad hoc simulations strongly depend on the choice of parameters, and they become less informative the less informed they are on current knowledge. Here, we have shown several important discrepancies between the used simulations and the current knowledge of the ToL and the eukaryogenesis period, which are relevant to test the effect of ghosts in some of the empirical studies that are claimed to be invalidated. Finally, even in the worst-case scenarios used by Tricou and colleagues, the number of trees supporting a correct ordering will be majoritarian. We therefore ask for caution when implying that a simulation falsifies a discovery based on empirical data.

## References

[1] Arnold BJ, Huang I-T, Hanage WP. Horizontal gene transfer and adaptive evolution in bacteria. Nat Rev Microbiol. 2021;20:206–218. doi: 10.1038/s41579-021-00650-4 34773098

[2] Gophna U, Altman-Price N. Horizontal Gene Transfer in Archaea-From Mechanisms to Genome Evolution. Annu Rev Microbiol. 2022;76:481–502. doi: 10.1146/annurev-micro-040820-124627 35667126

[3] Gabaldón T. Patterns and impacts of nonvertical evolution in eukaryotes: a paradigm shift. Ann N Y Acad Sci U S A. 2020:1476. doi: 10.1111/nyas.14471 32860228 PMC7589212

[4] Douglas GM, Langille MGI. Current and Promising Approaches to Identify Horizontal Gene Transfer Events in Metagenomes. Genome Biol Evol. 2019;11:2750–2766. doi: 10.1093/gbe/evz184 31504488 PMC6777429

[5] Tricou T, Tannier E, de Vienne DM. Ghost lineages can invalidate or even reverse findings regarding gene flow. PLoS Biol. 2022;20:e3001776. doi: 10.1371/journal.pbio.3001776 36103518 PMC9473628

[6] Pittis AA, Gabaldón T. Late acquisition of mitochondria by a host with chimaeric prokaryotic ancestry. Nature. 2016;531:101–104. doi: 10.1038/nature16941 26840490 PMC4780264

[7] Vosseberg J, van Hooff JJE, Marcet-Houben M, van Vlimmeren A, van Wijk LM, Gabaldón T, et al. Timing the origin of eukaryotic cellular complexity with ancient duplications. Nat Ecol Evol. 2021:5. doi: 10.1038/s41559-020-01320-z 33106602 PMC7610411

[8] Hahn MW, Hibbins MS. A Three-Sample Test for Introgression. Mol Biol Evol. 2019;36:2878–2882. doi: 10.1093/molbev/msz178 31373630

[9] Hug LA, Baker BJ, Anantharaman K, Brown CT, Probst AJ, Castelle CJ, et al. A new view of the tree of life. Nat Microbiol. 2016;1:16048. doi: 10.1038/nmicrobiol.2016.48 27572647

[10] Williams TA, Cox CJ, Foster PG, Szöllősi GJ, Embley TM. Phylogenomics provides robust support for a two-domains tree of life. Nat Ecol Evol. 2020;4:138–147. doi: 10.1038/s41559-019-1040-x 31819234 PMC6942926

[11] Coleman GA, Davín AA, Mahendrarajah TA, Szánthó LL, Spang A, Hugenholtz P, et al. A rooted phylogeny resolves early bacterial evolution. Science. 2021:372. doi: 10.1126/science.abe0511 33958449

[12] Susko E, Steel M, Roger AJ. Conditions under which distributions of edge length ratios on phylogenetic trees can be used to order evolutionary events. J Theor Biol. 2021;526:110788. doi: 10.1016/j.jtbi.2021.110788 34097914

[13] Fontaine MC, Pease JB, Steele A, Waterhouse RM, Neafsey DE, Sharakhov IV, et al. Mosquito genomics. Extensive introgression in a malaria vector species complex revealed by phylogenomics. Science. 2015;347:1258524. doi: 10.1126/science.1258524 25431491 PMC4380269

[14] Rabosky DL, Lovette IJ. Explosive evolutionary radiations: decreasing speciation or increasing extinction through time? Evolution. 2008;62:1866–1875. doi: 10.1111/j.1558-5646.2008.00409.x 18452577

